# Disseminated *Scedosporium apiospermum* central nervous system infection after lung transplantation: A case report with successful recovery

**DOI:** 10.1016/j.mmcr.2019.03.003

**Published:** 2019-03-16

**Authors:** Juuso Paajanen, Maija Halme, Maarit Palomäki, Veli-Jukka Anttila

**Affiliations:** a Department of Pulmonary Medicine, Heart and Lung Center, University of Helsinki and Helsinki University Hospital, Finland; b Department of Radiology, University of Helsinki and Helsinki University Hospital, Finland; c Department of Infectious Diseases, Inflammation Center, University of Helsinki and Helsinki University Hospital, Finland

**Keywords:** Cystic fibrosis, Lung transplantation, *Scedosporium apiospermum*, Disseminated CNS infection, Voriconazole

## Abstract

*Scedosporium* species are fungal opportunistic pathogens frequently seen in chronic lung diseases such as in cystic fibrosis (CF). They can cause a wide spectrum of diseases mainly in immunodeficient patients. Invasive, disseminated infections with poor prognosis have been described after lung transplantation. We present a CF-patient with disseminated *Scedosporium apiospermum* infection after lung transplantation. The patient had skin, surgical wound, spinal cord, and brain involvements. She recovered fully after prolonged course of voriconazole treatment.

## 1 Introduction

Cystic fibrosis (CF) is an inherited disease due to mutations in the cystic fibrosis transmembrane conductance regulator (CFTR) gene [1]. Defective CFTR protein causes abnormal ion transport across the apical surfaces of epithelia in multiple organ systems. One consequence of abnormal ion transport in the lung is dehydration and thickening of airway secretions. The disease is characterized by recurrent bacterial and fungal infections and progressive respiratory failure. Lung transplantation (LT) is a therapeutic option for patients with end-stage lung disease.

*Scedosporium* is a saprophytic fungus isolated from soil, polluted water and plant residues worldwide. The genus *Scedosporium* consists of three medically important species: *Scedosporium apiospermum (S.apiospermum), Scedosperium boydii (*formerly *Pseudoallescheria boydii)* and *Scedosporium aurantiacum* [2]*. Scedosporium* spp. is the second most prevalent opportunistic fungus after *Aspergillus* spp. found to colonize chronic lung diseases such as CF [3]. The role and pathogenicity of *S. apiospermum* in lung diseases is controversial, but either local or disseminated infections are described in immunodeficient patients [4]. Especially after organ transplant, the colonization may develop into invasive, disseminated infection with central nervous system (CNS) involvement leading to dismal outcome [5]. Thus, *Scedosporium* colonization prior to LT is considered as a contra-indication in some transplantation centers [3]. Unlike *Aspergillus*, *Scedosporium* spp. is inherently resistant to many antifungals such as amphotericin B and echinocandins. Voriconazole used alone or in combination is reported to be the most active agent against *Scedosporium* [6]. Also, a reduction in immunosuppression or surgical drainage should be considered when suitable.

Here, we present the first successfully treated disseminated *S. apiospermum* CNS infection after a lung transplantation.

## 2 Case

An 18-year-old woman with CF was considered as a candidate for bilateral LT. Her respiratory failure had advanced, so that a supplemental oxygen therapy and night-time non-invasive ventilation were initiated. Bilateral pneumothoraxes with subcutaneous emphysema were detected on an elective control in March 2015. At that time, her FEV1 had decreased to 1.35 L (34% of predicted). She was referred to the respiratory department where her ventilatory failure acutely progressed. After a short resuscitation she was connected to ventilator and subsequently to extracorporeal membrane oxygenation (ECMO). She was listed for a Scandinavian emergency LT. *S. apiospermum* was detected in fungal culture of the tracheal aspirate with susceptibility testing showing minimal inhibitory concentrations of: voriconazole 0.125mg/L, itraconazole 6mg/L, posaconazole 6mg/L and amphotericin B 12mg/L. After five days on ECMO, she underwent a bilateral LT (defined as day 0). *Pseudomonas aeruginosa* and *S. apiospermum* colonizations were detected in the extracted native lungs. The peri-operative course was complicated by *pseudomonas* septicemia which was treated with intravenous (IV) tazobactam/piperacillin, tobramycin, and oral ciprofloxacin with a good clinical outcome. IV caspofungin was started postoperatively for antifungal prophylaxis with a single loading dose of 70mg, followed by 50mg daily for 17 days.

Her baseline immunosuppression regimen consisted of tacrolimus, mycophenolate mofetil, and prednisolone. Prophylactic valganciclovir, azithromycin, trimethoprim/sulfamethoxazole and nebulized colistin and amphotericin B were initiated.

The first postoperative bronchoscopy at day 30 postoperatively revealed normal anastomotic healing process and otherwise unremarkable endobronchial findings. Bacterial and fungal cultures of bronchoalveolar lavage (BAL) were negative. Histological acute minimal rejection (A1B1) was detected in transbronchial lung biopsy (TBB). The patient was not treated with additional corticosteroids considering her good clinical condition and previous infections and colonizations.

**Fig. 1:**
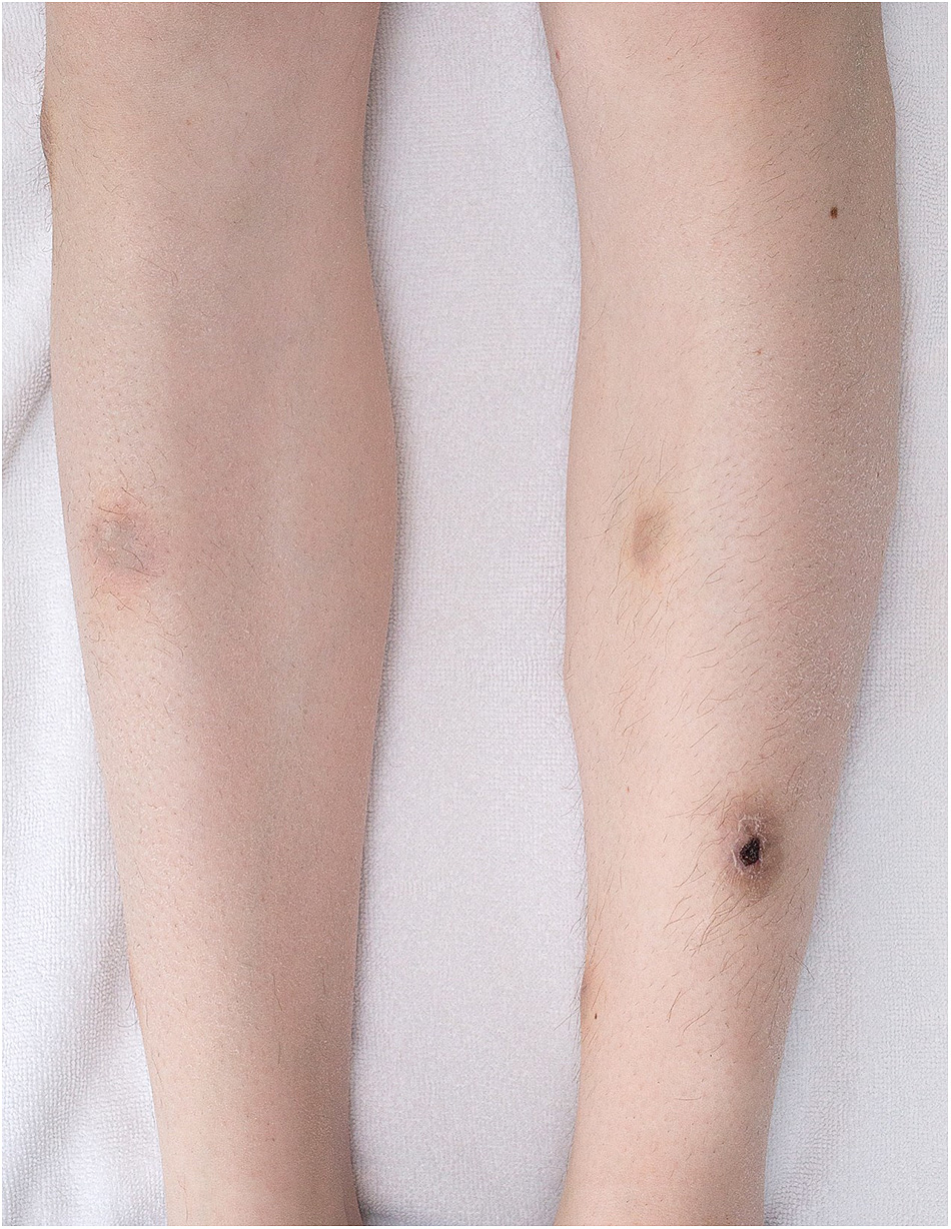
A photograph of the patient's lower extremity: slightly pigmented nodules sized one to two cm in diameter with small ulcerations is shown. The histological finding was septal panniculitis with fungal culture and nucleic acid test positive for *S. apiospermum.*

**Fig. 2:**
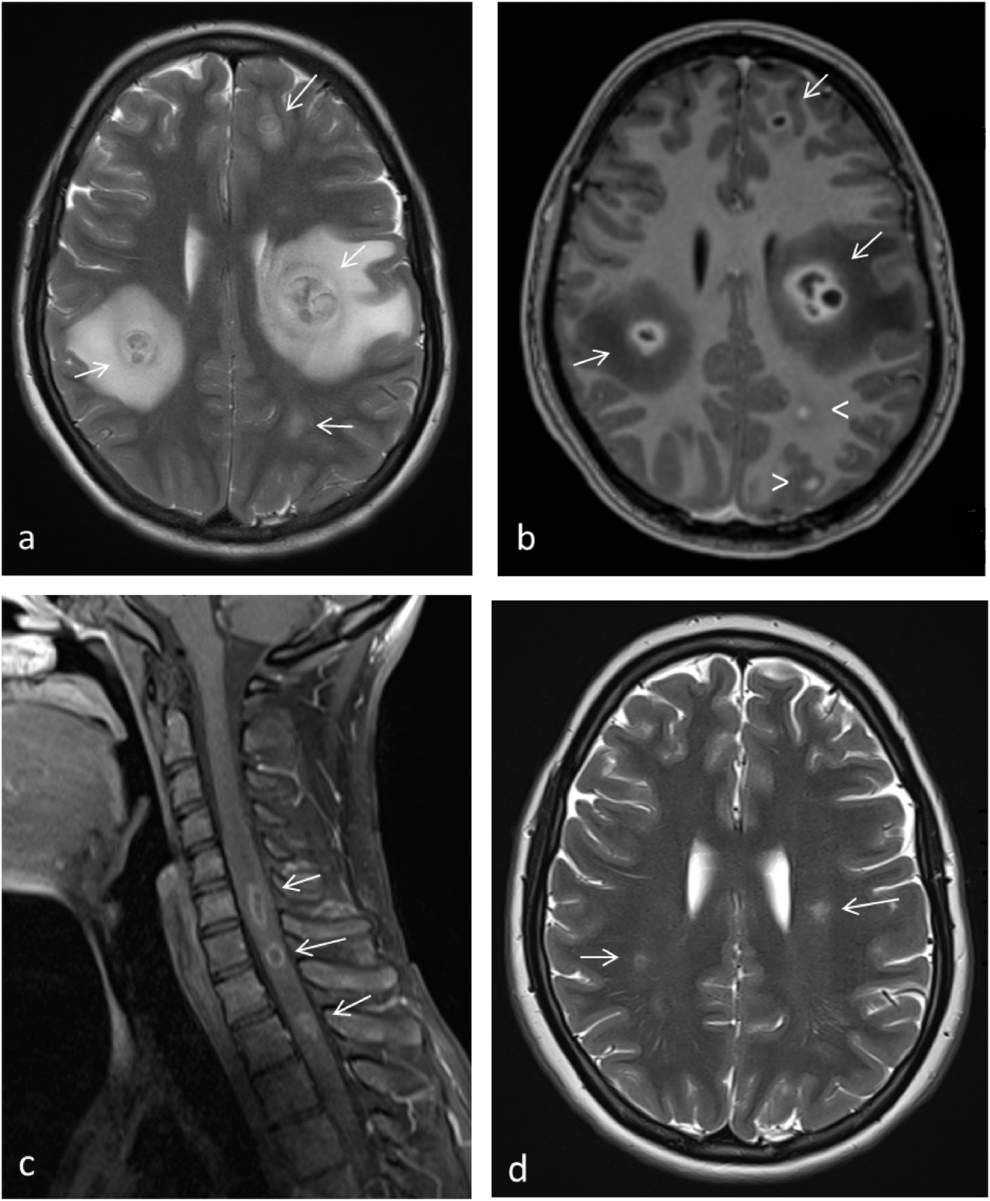
a–d: MRI T2 weighted imaging revealed multiple lesions with perifocal oedema (arrows) (a). Most of the lesions had ring-enhancement with gadolinium compatible with abscess (arrows). There were also nodular enhancing lesions (arrowheads) (b). Spinal cord MRI shows multiple ring-enhancing lesions (arrows) (c). The latest control image shows only small residual T2-lesions (arrows) that had remained stable over 8 months after completion of 35 months of voriconazole treatment (d).

At the second control visit 60 days postoperatively, she presented with upper back pain that radiated to the left leg. Symmetric, painful, palpable, and slightly pigmented nodules with small ulcerations had appeared on both legs (Fig. 1). The surgical wound had started to secrete. She had no fever. Her C-reactive protein was 31 mg/l, erythrocyte sedimentation rate 58 mm/h and leukocyte level 9.8 E9/l. Systemic fungal infection was suspected, and IV voriconazole was initiated at 300mg twice a day for 24 hours and at 200mg twice a day thereafter. At that time, her immunosuppression consisted of prednisolone, tacrolimus and mycophenolate mofetil. The latter was discontinued due to suspicion of systemic infection. Control bronchoscopy with TBB revealed no histological signs of rejection. In the following days, she developed neurological symptoms: headache, nausea, vertigo and her right side of the mouth was slightly dropped. Also, her left leg was numb, and the muscular strength was decreased. Computerized tomography showed no infiltrates in the lung parenchyma but there were small subcutaneous collections under the sternotomy. Brain magnetic resonance imaging (MRI) revealed multiple ring-enhancing lesions with perifocal oedema compatible with abscesses (Fig. 2a and b). Spine MRI showed oedema and multiple abscesses as well in cervical and thoracic spinal cord (Fig. 2c). Subsequently, the histopathological findings from initial skin nodule revealed septal panniculitis with fungal culture and nucleic acid test positive for *S. apiospermum. S. apiospermum* was also cultured from BAL fluid and the surgical wound secretion. Multiple blood cultures were taken with no signs of fungemia. We refrained from CNS biopsy due to consisted cultural findings matching with radiological appearance.

Neurological symptoms vanished gradually, and the patient was discharged from the hospital after three weeks of treatment. Intravenous voriconazole was continued at home for ten months. Miltefosine was combined to the therapy one month after initiation of voriconazole but it was discontinued after two days due to nausea, interactions with tacrolimus and on the other hand good clinical response to voriconazole. MRIs and clinical status were controlled every one to four months. After ten months of intravenous administration voriconazole was switched to oral tablets with a dose of 300mg twice daily. Therapeutic serum levels were confirmed by repeated measurements (target therapeutic limits 2–5.5mg/l). At a control visit in June 2018 three years after LT and 35 months of treatment, the voriconazole treatment was terminated. After the discontinuation of antifungal treatment, the patient has visited our clinic for two controls with no signs of fungal re-infection. The last visit was in January 2019, nearly four years after the LT and eight months after the termination of voriconazole. She was symptomless with preserved lung allograft function: Her FEV1 was 2.7 L (77% predicted). Brain MRI revealed only few residual lesions (Fig. 2d), which had been stable and inactive for months.

## 3 Discussion

To the best of our knowledge, the presented case is the first published *S. apiospermum* disseminated infection with brain and spinal cord abscesses after LT leading to a full recovery Previous reports have shown a good response to antifungals with or without surgical drainage in patients with *S. apiospermum* local spondylodiscitis, osteomyelitis, septic arthritis, or lung infections in LT recipients [7,8]. However, the case reports after LT on both disseminated and CNS involvement have been disappointing [5,[9], [10], [11], [12], [13], [14]]. Previously reported disseminated *S. apiospermum* infections after LT are reviewed in Table 1.

**Table 1:** Clinical characteristics of previously reported patients of disseminated Scedosporium apiospermum infections after lung transplantation.

Age, years	Sex	Antifungal prophylaxis	Time to diagnosis after LT	Infection sites	Antifungal therapy	Outcome (Survival time after diagnosis)	Reference
43	M	ITC	18 months	Pulmonary, mediastinum, joint, vertebra	ITC, CAS, AMB	Death (13 months)	[13]
57	F	ITC	14 months	Pulmonary, brain, breast implant, skin	VRC, TRB, POS	Death (shortly after diagnosis)	[13]
19	F	VRC	1 month	Eye, skin, mediastinum, chest wall, pulmonary, sinus, joint, vertebra	VRC, CAS, TRB, POS, AMB, PEN	Death (14 months)	[13]
20	F	None	11 months	Kidneys, eye, pulmonary, vertebra	NA	Death (5 months)	[3]
37	F	None	2 months	Pulmonary, brain, heart, eye	ITC, AMB	Death (1 month)	[3]
64	F	None	3 years	Pulmonary, septicemia, heart	AMB, ITC	Death (18 days)	[11]
37	F	VRC	2 months	Skin, brain, septicemia, heart	VRC, CAS, TRB	Death (6 months)	[14]
24	F	None	7.5 months	Heart, spleen, kidneys, brain	ITC, MIC	Death (1 month)	[14]
30	M	None	2 weeks	Pulmonary, heart	AMB, MIC	Death (7 days)	[10]
26	F	ITC, AMB	3 weeks	Skin, eye, brain	VRC, MIC	Death (6 months)	[9]
33	F	AMB, ITC, CAS	3 months	Joint, pulmonary	VRC	Alive	[8]
27	M	None	6 weeks	Brain, pulmonary	AMB	Death (shortly after diagnosis)	[8]
27	M	VRC, AMB	1 month	Pulmonary, heart, septic thrombus	VRC, TRB, CAS, POS, ANF, MTF	Death (7 months)	[12]

LT, lung transplantation; M, male; ITC, itraconazole; CAS, caspofungin; AMB, amphotericin B; F, female; VRC, voriconazole; TRB, terbinafine; POS, posaconazole; PEN, pentamidine; NA, Not available; MIC, miconazole; ANF, anidulafungin; MTF, miltefosine.

Careful balance in immunosuppression is needed to successfully manage patients after LT to prevent and treat both the rejection of the lung allograft and bacterial, viral, and fungal infections. Although less frequent than bacterial and viral infections, invasive fungal infection is associated with higher morbidity and mortality after LT [15]. The depth of immunosuppression is associated with both increased incidence and worse outcome of invasive fungal infections [5,15].

There is no widely accepted optimal recommendation for antifungal prophylaxis after LT [15]. The standard regimen used in our institute is trimethoprim/sulfamethoxazole for *Pneumocystis jirovecii*. In selected high-risk patients for *Aspergillus* infection, we have used nebulized amphotericin B and short-term systemic caspofungin prophylaxis with low invasive *Aspergillus* infection incidence [16]. However, this regimen has no effect in *S. apiospermum.* Several positive reports with either itraconazole, posaconazole or voriconazole prophylaxis have been reported in *S. apiospermum* colonization, even if the optimal dose or length of treatment are not well known [3,13]. In contrast, there are also reports with fatal invasive *Scedosporium* infections in spite of long-term voriconazole prophylaxis [13,14]. In our case, we didn't use any prophylactic antifungal targeted to *Scedosporium* before LT. After LT, the patient received inhaled amphotericin B among other prophylactic agents without measurable prophylactic effects. The use of prophylactic triazole should be considered for the first months after LT or in the event of temporary additional immunosuppression in high-risk patients for *Scedosporium* infections.

The exact dosage, duration or combination of antimycotic therapies in *Scedosporium* infections are not well known due to the lack of prospective studies. A successful therapeutic response in 57% of patients and a median survival time of 133 days were reported in a retrospective study of 107 patients with *Scedosporium* infections treated with voriconazole [17]. The median duration of the treatment was 103 days (range 1–802 days), while 21% of patients received treatment for a year or more. The initial treatment was similar to our case: intravenous 6mg/kg twice a day for one day, followed by 4mg/kg twice a day after switching to oral therapy. In another report of an LT patient, an initial response was seen in a disseminated *S. apiospermum* infection with ocular, skin and cerebrospinal fluid involvement [9]. However, a fatal relapse was seen only two days after the discontinuation of a six-month treatment period. In our case, we think that the immediate initiation of voriconazole was important for the good outcome. We used prolonged intravenous voriconazole regimen for ten months. The main reasons for that were slow recovery seen in MRI images, lack of side effects, and fear of inadequate therapeutic levels due to CF-related malabsorption. Mild photosensitivity reaction was the only adverse event reported by the patient. In hindsight, an earlier switch to oral treatment with voriconazole and repeated concentration controls could have been possible. We added miltefosine as combination therapy based on previously published *in vitro* susceptibility testing, but the treatment was terminated due to unwanted side effects and good clinical response to voriconazole [18].

In conclusion, we reported a lung transplant patient with disseminated *S. apiospermum* infection with CNS manifestation leading to a good and rapid response to voriconazole. Considering the preceding evidence in the literature, we think that prior colonization of *Scedosporium* should not be an absolute contraindication for a lung transplant. However, in the absence of prospective clinical trials, a careful case-by-case evaluation is needed to prevent and treat disseminated diseases. Especially the role of prophylactic antifungal therapy, preoperative clearance of potential reservoirs (e.g. sinuses), use of surgical drainage, and reduced immunotherapy will be needed to consider when treating LT patients with *Scedosporium* spp. colonization and infection.

## Conflict of interest

There are none.
